# Diaqua­bis­(4-meth­oxy­benzoato-κ*O* ^1^)bis­(nicotinamide-κ*N* ^1^)cobalt(II) dihydrate

**DOI:** 10.1107/S1600536810026462

**Published:** 2010-07-10

**Authors:** Tuncer Hökelek, Hakan Dal, Barış Tercan, Erdinç Tenlik, Hacali Necefoğlu

**Affiliations:** a Department of Physics, Hacettepe University, 06800 Beytepe, Ankara, Turkey; b Department of Chemistry, Faculty of Science, Anadolu University, 26470 Yenibağlar, Eskişehir, Turkey; c Department of Physics, Karabük University, 78050 Karabük, Turkey; d Department of Chemistry, Kafkas University, 36100 Kars, Turkey

## Abstract

In the mononuclear title compound, [Co(C_8_H_7_O_3_)_2_(C_6_H_6_N_2_O)_2_(H_2_O)_2_]·2H_2_O, the Co^II^ ion is located on a crystallographic inversion center. The asymmetric unit is completed by one 4-meth­oxy­benzoate anion, one nicotinamide (NA) ligand and one coordinated and one uncoordinated water mol­ecule. All ligands act in a monodentate mode. The four O atoms in the equatorial plane around the Co^II^ ion form a slightly distorted square-planar arrangement, while the slightly distorted octa­hedral coordination is completed by the two pyridine N atoms of the NA ligands in the axial positions. The dihedral angle between the carboxyl­ate group and the attached benzene ring is 6.47 (7)°, while the pyridine and benzene rings are oriented at a dihedral angle of 72.80 (4)°. An O—H⋯O hydrogen bond links the uncoordinated water mol­ecule to one of the carboxyl­ate groups. In the crystal structure, inter­molecular O—H⋯O, N—H⋯O and C—H⋯O hydrogen bonds link the mol­ecules into a three-dimensional network.

## Related literature

For niacin, see: Krishnamachari (1974 ▶). For *N*,*N*-diethyl­nicotinamide, see: Bigoli *et al.* (1972 ▶). For related structures, see: Hökelek *et al.* (1996 ▶, 2009*a* ▶,*b* ▶,*c* ▶); Hökelek & Necefoğlu (1998 ▶); Necefoğlu *et al.* (2010 ▶).
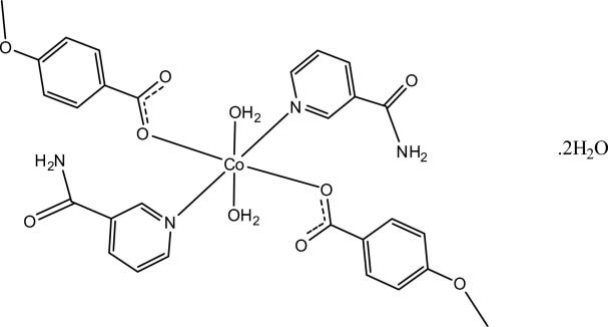


## Experimental

### 

#### Crystal data

[Co(C_8_H_7_O_3_)_2_(C_6_H_6_N_2_O)_2_(H_2_O)_2_]·2H_2_O
*M* *_r_* = 677.52
Triclinic, 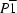

*a* = 8.1568 (2) Å
*b* = 9.7502 (2) Å
*c* = 10.0700 (3) Å
α = 101.151 (3)°
β = 91.796 (2)°
γ = 106.043 (3)°
*V* = 752.09 (4) Å^3^
*Z* = 1
Mo *K*α radiation
μ = 0.64 mm^−1^
*T* = 100 K
0.45 × 0.29 × 0.24 mm

#### Data collection

Bruker Kappa APEXII CCD area-detector diffractometer
Absorption correction: multi-scan (*SADABS*; Bruker, 2005 ▶) *T*_min_ = 0.799, *T*_max_ = 0.856
13470 measured reflections
3776 independent reflections
3541 reflections with *I* > 2σ(*I*)
*R*_int_ = 0.023

#### Refinement

*R*[*F* ^2^ > 2σ(*F* ^2^)] = 0.027
*wR*(*F* ^2^) = 0.074
*S* = 1.07
3776 reflections
230 parameters
H atoms treated by a mixture of independent and constrained refinement
Δρ_max_ = 0.55 e Å^−3^
Δρ_min_ = −0.31 e Å^−3^

### 

Data collection: *APEX2* (Bruker, 2007 ▶); cell refinement: *SAINT* (Bruker, 2007 ▶); data reduction: *SAINT*; program(s) used to solve structure: *SHELXS97* (Sheldrick, 2008 ▶); program(s) used to refine structure: *SHELXL97* (Sheldrick, 2008 ▶); molecular graphics: *ORTEP-3 for Windows* (Farrugia, 1997 ▶); software used to prepare material for publication: *WinGX* (Farrugia, 1999 ▶) and *PLATON* (Spek, 2009 ▶).

## Supplementary Material

Crystal structure: contains datablocks I, global. DOI: 10.1107/S1600536810026462/cv2740sup1.cif

Structure factors: contains datablocks I. DOI: 10.1107/S1600536810026462/cv2740Isup2.hkl

Additional supplementary materials:  crystallographic information; 3D view; checkCIF report

## Figures and Tables

**Table 1:** Hydrogen-bond geometry (Å, °)

*D*—H⋯*A*	*D*—H	H⋯*A*	*D*⋯*A*	*D*—H⋯*A*
N2—H21⋯O6^i^	0.876 (19)	1.969 (19)	2.8343 (15)	169.6 (17)
O5—H51⋯O4^ii^	0.841 (19)	1.868 (19)	2.6976 (13)	168.6 (19)
O5—H52⋯O2^iii^	0.817 (19)	1.924 (19)	2.7064 (13)	159.8 (19)
O6—H61⋯O2	0.81 (3)	2.10 (3)	2.9009 (14)	170 (2)
O6—H62⋯O2^iv^	0.84 (2)	1.97 (2)	2.8068 (14)	173.9 (19)
C4—H4⋯O4^v^	0.95	2.59	3.4225 (16)	146
C9—H9⋯O1^iii^	0.95	2.40	3.0325 (16)	124
C10—H10⋯O5^vi^	0.95	2.40	3.2925 (17)	156

Symmetry codes: (i) 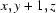; (ii) 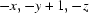; (iii) 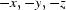; (iv) 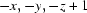; (v) 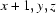; (vi) 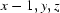.

**Fig. 1.**
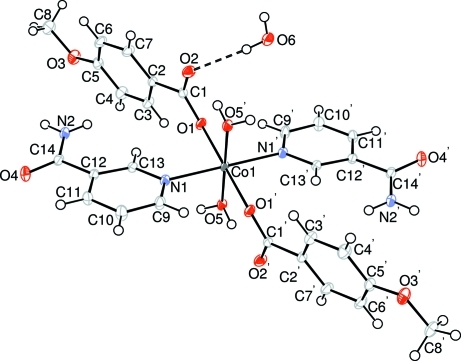
The molecular structure of the title compound with the atom-numbering scheme. Displacement ellipsoids are drawn at the 50% probability level. Primed atoms are generated by the symmetry operator: (') -x, -y, -z. Dashed lines indicate the hydrogen-bonding.

## supplementary crystallographic information

### Comment

As a part of our ongoing investigation on transition metal complexes of nicotinamide (NA), one form of niacin (Krishnamachari, 1974), and/or the nicotinic acid derivative *N*,*N*-diethylnicotinamide (DENA), an important respiratory stimulant (Bigoli *et al.*, 1972), the title compound was synthesized and its crystal structure is reported herein.

The title compound, (I), is a mononuclear complex, where the Co^II^ ion is located on a crystallographic inversion center. The asymmetric unit contains one 4-methoxybenzoate (PMOB) anion, one nicotinamide (NA) ligand and one coordinated and one uncoordinated water molecules, all ligands are monodentate (Fig. 1). The crystal structures of some NA and/or DENA complexes of Cu^II^, Co^II^, Ni^II^, Mn^II^ and Zn^II^ ions, [Cu(C_7_H_5_O_2_)_2_(C_10_H_14_N_2_O)_2_], (II) (Hökelek *et al.*, 1996), [Co(C_6_H_6_N_2_O)_2_(C_7_H_4_NO_4_)_2_(H_2_O)_2_], (III) (Hökelek & Necefoğlu, 1998), [Ni(C_7_H_4_ClO_2_)_2_(C_6_H_6_N_2_O)_2_(H_2_O)_2_], (IV) (Hökelek *et al.*, 2009*a*), [Ni(C_8_H_7_O_2_)_2_(C_6_H_6_N_2_O)_2_(H_2_O)_2_], (V) (Necefoğlu *et al.*, 2010), [Mn(C_7_H_4_ClO_2_)_2_(C_10_H_14_N_2_O)_2_(H_2_O)_2_], (VI) (Hökelek *et al.*, 2009*b*) and [Zn(C_7_H_4_BrO_2_)_2_(C_6_H_6_N_2_O)_2_(H_2_O)_2_], (VII) (Hökelek *et al.*, 2009*c*) have also been reported. In (II), two benzoate ions are coordinated to the Cu atom as bidentate ligands, while in other structures all ligands being monodentate.

The four O atoms (O1, O5, and the symmetry-related atoms, O1', O5') in the equatorial plane around the Co^II^ ion form a slightly distorted square-planar arrangement, while the slightly distorted octahedral coordination is completed by the two N atoms of the NA ligands (N1, N1') in the axial positions (Fig. 1). The near equality of the C1—O1 [1.2698 (14) Å] and C1—O2 [1.2626 (15) Å] bonds in the carboxylate group indicates a delocalized bonding arrangement, rather than localized single and double bonds. The average Co—O bond length is 2.0895 (9) Å, and the Co^II^ ion is displaced out of the least-squares plane of the carboxylate group (O1/C1/O2) by 0.8407 (1) Å. The dihedral angle between the planar carboxylate group and the benzene ring A (C2—C7) is 6.47 (7)°, while that between rings A and B (N1/C9—C13) is 72.80 (4)°. An intramolecular O—H···O hydrogen bond (Table 1) links the uncoordinated water molecule to one of the carboxylate groups (Fig. 1).

In the crystal structure, intermolecular O—H···O, N—H···O and C—H···O hydrogen bonds (Table 1) link the molecules into a three-dimensional network.

### Experimental

The title compound was prepared by the reaction of CoSO_4_.7H_2_O (2.81 g, 10 mmol) in H_2_O (50 ml) and nicotinamide (2.44 g, 20 mmol) in H_2_O (50 ml) with sodium 4-methoxybenzoate (3.48 g, 20 mmol) in H_2_O (100 ml). The mixture was filtered and set aside to crystallize at ambient temperature for one week, giving pink single crystals.

### Refinement

Atoms H21, H22 (for NH_2_) and H51, H52, H61, H62 (for H_2_O) were located in a difference Fourier map and refined isotropically. The remaining H atoms were positioned geometrically with C—H = 0.95 and 0.98 Å for aromatic and methyl H atoms and constrained to ride on their parent atoms, with *U*_iso_(H) = *xU*_eq_(C), where *x* = 1.5 for methyl H and *x* = 1.2 for aromatic H atoms.

### Crystal data

**Table:**

[Co(C_8_H_7_O_3_)_2_(C_6_H_6_N_2_O)_2_(H_2_O)_2_]·2H_2_O	*Z* = 1
*M**_r_* = 677.52	*F*(000) = 353
Triclinic, *P*1	*D*_x_ = 1.496 Mg m^−^^3^
Hall symbol: -P 1	Mo *K*α radiation, λ = 0.71073 Å
*a* = 8.1568 (2) Å	Cell parameters from 9220 reflections
*b* = 9.7502 (2) Å	θ = 2.6–28.4°
*c* = 10.0700 (3) Å	µ = 0.64 mm^−^^1^
α = 101.151 (3)°	*T* = 100 K
β = 91.796 (2)°	Block, pink
γ = 106.043 (3)°	0.45 × 0.29 × 0.24 mm
*V* = 752.09 (4) Å^3^	

### Data collection

**Table:**

Bruker Kappa APEXII CCD area-detector diffractometer	3776 independent reflections
Radiation source: fine-focus sealed tube	3541 reflections with *I* > 2σ(*I*)
graphite	*R*_int_ = 0.023
φ and ω scans	θ_max_ = 28.5°, θ_min_ = 2.1°
Absorption correction: multi-scan (*SADABS*; Bruker, 2005)	*h* = −10→10
*T*_min_ = 0.799, *T*_max_ = 0.856	*k* = −13→10
13470 measured reflections	*l* = −13→13

### Refinement

**Table:**

Refinement on *F*^2^	Primary atom site location: structure-invariant direct methods
Least-squares matrix: full	Secondary atom site location: difference Fourier map
*R*[*F*^2^ > 2σ(*F*^2^)] = 0.027	Hydrogen site location: inferred from neighbouring sites
*wR*(*F*^2^) = 0.074	H atoms treated by a mixture of independent and constrained refinement
*S* = 1.07	*w* = 1/[σ^2^(*F*_o_^2^) + (0.0387*P*)^2^ + 0.2836*P*] where *P* = (*F*_o_^2^ + 2*F*_c_^2^)/3
3776 reflections	(Δ/σ)_max_ < 0.001
230 parameters	Δρ_max_ = 0.55 e Å^−^^3^
0 restraints	Δρ_min_ = −0.31 e Å^−^^3^

### Special details

**Table:**

Geometry. All esds (except the esd in the dihedral angle between two l.s. planes) are estimated using the full covariance matrix. The cell esds are taken into account individually in the estimation of esds in distances, angles and torsion angles; correlations between esds in cell parameters are only used when they are defined by crystal symmetry. An approximate (isotropic) treatment of cell esds is used for estimating esds involving l.s. planes.
Refinement. Refinement of F^2^ against ALL reflections. The weighted R-factor wR and goodness of fit S are based on F^2^, conventional R-factors R are based on F, with F set to zero for negative F^2^. The threshold expression of F^2^ > 2sigma(F^2^) is used only for calculating R-factors(gt) etc. and is not relevant to the choice of reflections for refinement. R-factors based on F^2^ are statistically about twice as large as those based on F, and R- factors based on ALL data will be even larger.

### Fractional atomic coordinates and isotropic or equivalent isotropic displacement parameters (Å^2^)

**Table:**

	*x*	*y*	*z*	*U*_iso_*/*U*_eq_	
Co1	0.0000	0.0000	0.0000	0.01140 (7)	
O1	0.16134 (11)	0.15267 (9)	0.15761 (8)	0.01516 (17)	
O2	0.02618 (12)	0.12855 (9)	0.34523 (9)	0.02010 (19)	
O3	0.49079 (13)	0.79361 (10)	0.49240 (10)	0.0241 (2)	
O4	−0.23377 (11)	0.62299 (9)	0.11798 (9)	0.01950 (19)	
O5	0.16105 (11)	0.08580 (10)	−0.14044 (9)	0.01498 (17)	
H51	0.180 (2)	0.174 (2)	−0.145 (2)	0.038 (5)*	
H52	0.118 (2)	0.034 (2)	−0.214 (2)	0.034 (5)*	
O6	0.10074 (14)	−0.13088 (11)	0.39872 (10)	0.0230 (2)	
H61	0.091 (3)	−0.056 (3)	0.380 (2)	0.046 (6)*	
H62	0.060 (3)	−0.137 (2)	0.474 (2)	0.044 (6)*	
N1	−0.15075 (13)	0.15172 (11)	−0.00112 (10)	0.01377 (19)	
N2	−0.00471 (14)	0.58909 (12)	0.22425 (11)	0.0175 (2)	
H21	0.028 (2)	0.680 (2)	0.2689 (18)	0.029 (4)*	
H22	0.050 (2)	0.530 (2)	0.2387 (17)	0.024 (4)*	
C1	0.13157 (15)	0.20178 (12)	0.27783 (11)	0.0141 (2)	
C2	0.22590 (15)	0.35792 (12)	0.33911 (12)	0.0141 (2)	
C3	0.35076 (16)	0.43797 (14)	0.26923 (12)	0.0181 (2)	
H3	0.3764	0.3923	0.1834	0.022*	
C4	0.43723 (16)	0.58247 (14)	0.32337 (13)	0.0203 (3)	
H4	0.5229	0.6350	0.2755	0.024*	
C5	0.39860 (16)	0.65132 (13)	0.44861 (13)	0.0177 (2)	
C6	0.27348 (17)	0.57414 (14)	0.51879 (13)	0.0204 (3)	
H6	0.2457	0.6207	0.6034	0.024*	
C7	0.18914 (17)	0.42784 (14)	0.46397 (12)	0.0187 (2)	
H7	0.1050	0.3748	0.5127	0.022*	
C8	0.4554 (2)	0.87023 (15)	0.61895 (14)	0.0281 (3)	
H8A	0.5296	0.9710	0.6381	0.042*	
H8B	0.4770	0.8219	0.6917	0.042*	
H8C	0.3352	0.8703	0.6139	0.042*	
C9	−0.30977 (16)	0.09903 (13)	−0.06401 (13)	0.0179 (2)	
H9	−0.3478	−0.0009	−0.1087	0.021*	
C10	−0.42123 (16)	0.18327 (14)	−0.06690 (14)	0.0210 (3)	
H10	−0.5321	0.1423	−0.1140	0.025*	
C11	−0.36763 (16)	0.32850 (13)	0.00028 (13)	0.0173 (2)	
H11	−0.4412	0.3888	−0.0002	0.021*	
C12	−0.20437 (15)	0.38459 (12)	0.06844 (11)	0.0134 (2)	
C13	−0.10001 (15)	0.29263 (12)	0.06338 (11)	0.0132 (2)	
H13	0.0126	0.3314	0.1077	0.016*	
C14	−0.14729 (15)	0.54226 (13)	0.14024 (12)	0.0143 (2)	

### Atomic displacement parameters (Å^2^)

**Table:**

	*U*^11^	*U*^22^	*U*^33^	*U*^12^	*U*^13^	*U*^23^
Co1	0.01336 (11)	0.00782 (11)	0.01164 (11)	0.00330 (8)	−0.00119 (8)	−0.00122 (8)
O1	0.0164 (4)	0.0127 (4)	0.0143 (4)	0.0046 (3)	−0.0009 (3)	−0.0024 (3)
O2	0.0282 (5)	0.0123 (4)	0.0153 (4)	0.0002 (4)	0.0015 (3)	0.0004 (3)
O3	0.0276 (5)	0.0107 (4)	0.0266 (5)	−0.0011 (4)	−0.0033 (4)	−0.0028 (4)
O4	0.0210 (4)	0.0117 (4)	0.0268 (5)	0.0070 (3)	0.0020 (4)	0.0029 (3)
O5	0.0177 (4)	0.0099 (4)	0.0155 (4)	0.0030 (3)	−0.0010 (3)	−0.0002 (3)
O6	0.0356 (5)	0.0144 (5)	0.0177 (5)	0.0072 (4)	0.0029 (4)	0.0006 (4)
N1	0.0159 (5)	0.0109 (5)	0.0136 (4)	0.0043 (4)	−0.0002 (4)	0.0003 (4)
N2	0.0234 (5)	0.0114 (5)	0.0171 (5)	0.0067 (4)	−0.0006 (4)	−0.0006 (4)
C1	0.0166 (5)	0.0115 (5)	0.0135 (5)	0.0055 (4)	−0.0037 (4)	0.0000 (4)
C2	0.0169 (5)	0.0110 (5)	0.0134 (5)	0.0044 (4)	−0.0020 (4)	−0.0001 (4)
C3	0.0197 (6)	0.0153 (6)	0.0171 (5)	0.0041 (5)	0.0020 (4)	−0.0007 (4)
C4	0.0195 (6)	0.0162 (6)	0.0222 (6)	0.0016 (5)	0.0025 (5)	0.0023 (5)
C5	0.0192 (6)	0.0104 (5)	0.0198 (6)	0.0018 (4)	−0.0056 (4)	−0.0011 (4)
C6	0.0277 (6)	0.0140 (6)	0.0151 (5)	0.0034 (5)	0.0000 (5)	−0.0033 (4)
C7	0.0245 (6)	0.0133 (6)	0.0144 (5)	0.0014 (5)	0.0019 (4)	0.0000 (4)
C8	0.0387 (8)	0.0135 (6)	0.0246 (7)	0.0033 (6)	−0.0079 (6)	−0.0060 (5)
C9	0.0178 (6)	0.0115 (5)	0.0215 (6)	0.0038 (4)	−0.0030 (5)	−0.0017 (4)
C10	0.0164 (6)	0.0165 (6)	0.0279 (7)	0.0055 (5)	−0.0045 (5)	−0.0003 (5)
C11	0.0173 (5)	0.0152 (6)	0.0210 (6)	0.0080 (5)	0.0017 (4)	0.0031 (5)
C12	0.0168 (5)	0.0112 (5)	0.0125 (5)	0.0045 (4)	0.0031 (4)	0.0024 (4)
C13	0.0152 (5)	0.0111 (5)	0.0123 (5)	0.0033 (4)	0.0002 (4)	0.0008 (4)
C14	0.0185 (5)	0.0114 (5)	0.0134 (5)	0.0048 (4)	0.0053 (4)	0.0024 (4)

### Geometric parameters (Å, °)

**Table:**

Co1—O1	2.0831 (8)	C2—C7	1.3931 (16)
Co1—O1^i^	2.0831 (8)	C3—C4	1.3817 (17)
Co1—O5	2.0958 (9)	C3—H3	0.9500
Co1—O5^i^	2.0958 (9)	C4—C5	1.3982 (17)
Co1—N1	2.1706 (10)	C4—H4	0.9500
Co1—N1^i^	2.1706 (10)	C5—C6	1.3907 (18)
O1—C1	1.2698 (14)	C6—C7	1.3935 (17)
O2—C1	1.2626 (15)	C6—H6	0.9500
O3—C5	1.3603 (15)	C7—H7	0.9500
O3—C8	1.4281 (17)	C8—H8A	0.9800
O4—C14	1.2381 (15)	C8—H8B	0.9800
O5—H51	0.84 (2)	C8—H8C	0.9800
O5—H52	0.81 (2)	C9—C10	1.3865 (17)
O6—H61	0.81 (2)	C9—H9	0.9500
O6—H62	0.84 (2)	C10—C11	1.3866 (17)
N1—C9	1.3423 (15)	C10—H10	0.9500
N1—C13	1.3433 (15)	C11—C12	1.3923 (17)
N2—C14	1.3312 (16)	C11—H11	0.9500
N2—H21	0.875 (19)	C12—C13	1.3921 (16)
N2—H22	0.854 (18)	C12—C14	1.5021 (16)
C1—C2	1.5000 (16)	C13—H13	0.9500
C2—C3	1.3999 (17)		
			
O1^i^—Co1—O1	180.00 (6)	C3—C4—C5	120.01 (11)
O1—Co1—O5	89.63 (3)	C3—C4—H4	120.0
O1^i^—Co1—O5	90.37 (3)	C5—C4—H4	120.0
O1—Co1—O5^i^	90.37 (3)	O3—C5—C4	115.24 (11)
O5—Co1—O5^i^	180.00 (5)	O3—C5—C6	124.82 (11)
O1^i^—Co1—O5^i^	89.63 (3)	C6—C5—C4	119.95 (11)
O1—Co1—N1	88.17 (3)	C5—C6—C7	119.43 (11)
O1^i^—Co1—N1	91.83 (3)	C5—C6—H6	120.3
O1—Co1—N1^i^	91.83 (3)	C7—C6—H6	120.3
O1^i^—Co1—N1^i^	88.17 (3)	C2—C7—C6	121.26 (11)
O5—Co1—N1	93.31 (4)	C2—C7—H7	119.4
O5^i^—Co1—N1	86.69 (4)	C6—C7—H7	119.4
O5—Co1—N1^i^	86.69 (4)	O3—C8—H8A	109.5
O5^i^—Co1—N1^i^	93.31 (4)	O3—C8—H8B	109.5
N1^i^—Co1—N1	180.00 (4)	O3—C8—H8C	109.5
C1—O1—Co1	130.19 (8)	H8A—C8—H8B	109.5
C5—O3—C8	117.77 (11)	H8A—C8—H8C	109.5
Co1—O5—H51	120.1 (13)	H8B—C8—H8C	109.5
Co1—O5—H52	104.5 (13)	N1—C9—C10	123.21 (11)
H52—O5—H51	109.5 (19)	N1—C9—H9	118.4
H62—O6—H61	107 (2)	C10—C9—H9	118.4
C9—N1—Co1	117.67 (8)	C9—C10—C11	118.68 (11)
C9—N1—C13	117.60 (10)	C9—C10—H10	120.7
C13—N1—Co1	124.61 (8)	C11—C10—H10	120.7
C14—N2—H21	119.2 (12)	C10—C11—C12	119.02 (11)
C14—N2—H22	120.4 (12)	C10—C11—H11	120.5
H22—N2—H21	120.2 (16)	C12—C11—H11	120.5
O1—C1—C2	116.55 (10)	C11—C12—C14	118.57 (10)
O2—C1—O1	124.00 (11)	C13—C12—C11	118.26 (11)
O2—C1—C2	119.43 (10)	C13—C12—C14	123.14 (11)
C3—C2—C1	120.15 (10)	N1—C13—C12	123.19 (11)
C7—C2—C1	121.38 (11)	N1—C13—H13	118.4
C7—C2—C3	118.45 (11)	C12—C13—H13	118.4
C2—C3—H3	119.6	O4—C14—N2	122.96 (11)
C4—C3—C2	120.90 (11)	O4—C14—C12	118.73 (11)
C4—C3—H3	119.6	N2—C14—C12	118.31 (10)
			
O5—Co1—O1—C1	158.01 (10)	O2—C1—C2—C3	176.09 (11)
O5^i^—Co1—O1—C1	−21.99 (10)	O2—C1—C2—C7	−5.62 (17)
N1—Co1—O1—C1	64.69 (10)	C1—C2—C3—C4	179.13 (11)
N1^i^—Co1—O1—C1	−115.31 (10)	C7—C2—C3—C4	0.78 (18)
O1—Co1—N1—C9	−163.03 (9)	C1—C2—C7—C6	−178.10 (11)
O1^i^—Co1—N1—C9	16.97 (9)	C3—C2—C7—C6	0.22 (19)
O1—Co1—N1—C13	12.88 (9)	C2—C3—C4—C5	−0.99 (19)
O1^i^—Co1—N1—C13	−167.12 (9)	C3—C4—C5—O3	−179.82 (11)
O5—Co1—N1—C9	107.45 (9)	C3—C4—C5—C6	0.19 (19)
O5^i^—Co1—N1—C9	−72.55 (9)	O3—C5—C6—C7	−179.20 (12)
O5—Co1—N1—C13	−76.64 (9)	C4—C5—C6—C7	0.79 (19)
O5^i^—Co1—N1—C13	103.36 (9)	C5—C6—C7—C2	−1.0 (2)
Co1—O1—C1—O2	31.88 (16)	N1—C9—C10—C11	−1.2 (2)
Co1—O1—C1—C2	−146.38 (8)	C9—C10—C11—C12	−0.15 (19)
C8—O3—C5—C4	179.22 (11)	C10—C11—C12—C13	1.50 (17)
C8—O3—C5—C6	−0.80 (19)	C10—C11—C12—C14	179.68 (11)
Co1—N1—C9—C10	177.21 (10)	C11—C12—C13—N1	−1.72 (17)
C13—N1—C9—C10	1.01 (18)	C14—C12—C13—N1	−179.81 (10)
Co1—N1—C13—C12	−175.45 (8)	C11—C12—C14—O4	−12.03 (16)
C9—N1—C13—C12	0.47 (17)	C11—C12—C14—N2	168.54 (11)
O1—C1—C2—C3	−5.57 (16)	C13—C12—C14—O4	166.05 (11)
O1—C1—C2—C7	172.73 (11)	C13—C12—C14—N2	−13.38 (17)

Symmetry codes: (i) −*x*, −*y*, −*z*.

### Hydrogen-bond geometry (Å, °)

**Table:**

*D*—H···*A*	*D*—H	H···*A*	*D*···*A*	*D*—H···*A*
N2—H21···O6^ii^	0.876 (19)	1.969 (19)	2.8343 (15)	169.6 (17)
O5—H51···O4^iii^	0.841 (19)	1.868 (19)	2.6976 (13)	168.6 (19)
O5—H52···O2^iv^	0.817 (19)	1.924 (19)	2.7064 (13)	159.8 (19)
O6—H61···O2	0.81 (3)	2.10 (3)	2.9009 (14)	170 (2)
O6—H62···O2^v^	0.84 (2)	1.97 (2)	2.8068 (14)	173.9 (19)
C4—H4···O4^vi^	0.95	2.59	3.4225 (16)	146
C9—H9···O1^iv^	0.95	2.40	3.0325 (16)	124
C10—H10···O5^vii^	0.95	2.40	3.2925 (17)	156

Symmetry codes: (ii) *x*, *y*+1, *z*; (iii) −*x*, −*y*+1, −*z*; (iv) −*x*, −*y*, −*z*; (v) −*x*, −*y*, −*z*+1; (vi) *x*+1, *y*, *z*; (vii) *x*−1, *y*, *z*.
